# Comparing two pre-operative anxiety scales for predicting postoperative pain in ambulatory surgery: Data from a multicentre observational study

**DOI:** 10.1097/EA9.0000000000000102

**Published:** 2026-03-30

**Authors:** Caroline S. Dahlem, Catarina T. Monteiro, Eunice Mendes, Carmen C. Oliveira, Margarida Bettencourt, Luis Azevedo, Joana Berger-Estilita, Cristina Granja

**Affiliations:** From the RISE-Health, Faculty of Medicine, University of Porto, Porto, Portugal (CSD, CCO, LA, JB-E, CG), Department of Anaesthesiology, Unidade Local de Saúde Entre Douro e Vouga, Santa Maria da Feira (CSD), Centro Académico Clínico Egas Moniz Health Alliance, University of Aveiro, Aveiro (CSD), Department of Anaesthesiology, Unidade Local de Saúde Vila Nova de Gaia/Espinho, Vila Nova de Gaia (CTM, CCO), Department of Anaesthesiology, Unidade Local de Saúde de Coimbra, Coimbra (EM), Department of Anaesthesiology, Unidade Local de Saúde do Baixo Vouga, EPE, Aveiro (MB), Department of Community Medicine, Information and Health Decision Sciences – MEDCIDS, Faculty of Medicine, University of Porto (FMUP), Porto, Portugal (LA), Institute for Medical Education, University of Bern, Bern, Switzerland (JB-E), Department of Anaesthesiology, Unidade Local de Saúde São João (CG), Department of Surgery and Physiology, Faculty of Medicine, University of Porto, Porto, Portugal (CG)

## Abstract

**BACKGROUND:**

Pre-operative anxiety is present in most day surgery patients and is associated with increased postoperative pain. However, most anaesthetists do not objectively evaluate pre-operative anxiety. Both Surgical Fear Questionnaire (SFQ) and Anxiety Numeric Rating Scale (ANRS) are tools that can be used for standardised anxiety assessment.

**OBJECTIVE:**

To compare the SFQ and ANRS in assessing pre-operative anxiety and predicting postoperative pain; to identify predictive factors for high pre-operative anxiety.

**STUDY DESIGN:**

Secondary analysis of a multicentre prospective observational study.

**SETTING:**

Three Portuguese ambulatory surgery units.

PATIENTS Two hundred and ninety-six patients underwent open inguinal hernia repair between September 2018 and March 2020.

**INTERVENTION:**

Patients were assessed for pre-operative anxiety using the SFQ and ANRS before surgery. Postoperative evaluations were conducted at 24 h, 7 days, 3 months and 1 year.

**MAIN OUTCOME MEASURES:**

Pre-operative anxiety and postoperative pain, Global Surgery Recovery Index and patient satisfaction.

**RESULTS:**

Two hundred and fifty-four patients completed the 12-month follow-up, with 260 men and 31 women analysed. The mean pre-operative SFQ score was 21.6 ± 15.8, with cutoffs of 20/24 for predicting acute/chronic pain. The mean pre-operative ANRS score was 3.7 ± 2.5, with an optimal cutoff of 4. Patients with SFQ at least 21 had worse postoperative outcomes at all time points (*P* < 0.03). The SFQ could predict pain at least 4 at all analysed time points while the ANRS could only predict pain 1 year after surgery. Independent predictors of high pre-operative anxiety included younger age, female sex, BMI less than 30 and pre-operative pain score at least 4.

**CONCLUSION:**

Pre-operative anxiety evaluation allows for the identification of patients at a high risk for postoperative complications. Both instruments demonstrated some ability to predict postoperative pain and might be valuable in the pre-operative setting, with the SFQ showing better performance.

**TRIAL REGISTRATION:**

NCT03499730

**KEY POINTS**

- The Surgical Fear Questionnaire (SFQ) was more effective than the Anxiety Numeric Rating Scale (ANRS) in predicting postoperative pain in ambulatory hernia surgery.
- Proposed cutoffs for pre-operative anxiety in this context would be 21 for the SFQ and 5 for the ANRS.
- Higher pre-operative anxiety was significantly associated with increased postoperative pain, reduced patient satisfaction and worse recovery.
- Independent predictors of high pre-operative anxiety included younger age, female sex, BMI less than 30 and pre-existing pain.
- Incorporating structured anxiety assessment tools like SFQ or ANRS may improve the identification of high-risk patients and support tailored peri-operative care.

## Introduction

Anxiety is a common but often underestimated issue in patients undergoing ambulatory surgery. According to Spielberger, it is ’a temporary emotional state marked by tension and apprehension, triggered by perceived threats and varying over time’.^1^ For many patients, anxiety is not just a passing concern; it is frequently reported as the most distressing part of the peri-operative experience.^2–5^

Pre-operative anxiety not only affects psychological well being but is also closely linked to acute and chronic postoperative pain, emphasising the importance of effective management.^6–8^ Recognising this, European guidelines now recommend pre-operative anxiety assessment.^9^ However, no standardised instrument or specific cutoff values have been established, leaving many anaesthesiologists to rely on subjective judgement.^9–11^ Studies suggest that this subjective approach may lead to inconsistencies, with many anaesthesiologists underestimating patient anxiety.^10,11^

### Anxiety assessment scales

Few instruments are available for assessing pre-operative anxiety,^12,13^ and some scales might be more suitable for specific medical conditions or patient populations.^14^ As a result, many studies rely on more general anxiety assessment tools, including the Hospital Anxiety and Depression Scale (HADS),^13,15^ the State-Trait Anxiety Inventory (STAI)^13^ or a Visual Analog Scale (VAS) measuring anxiety levels.^16,17^

Setting-specific validated tools such as the Surgical Fear Questionnaire (SFQ)^18^ and the Anxiety Numeric Rating Scale (ANRS)^19^ have been developed to address this gap. The SFQ evaluates pre-operative fear using two subscales: fear of short-term and long-term surgical outcomes. It has been validated for the Portuguese population.^18^ Meanwhile, simple rating scales such as the Numeric Rating Scale (NRS)^20^ and Visual Analog Scale (VAS)^21,22^ offer quick, reliable assessments of anxiety with strong correlations to the State-Trait Anxiety Inventory (STAI) and proven predictive validity for postoperative pain.^18,20–23^

This study aimed to compare the SFQ and ANRS in assessing pre-operative anxiety and predicting postoperative pain in a Portuguese ambulatory surgery population. In addition, we sought to identify key factors that contributed to high pre-operative anxiety, which could help optimise peri-operative care and improve surgical outcomes.

## Materials and methods

### Ethics

Ethical approval for this study was provided by the Ethical Committees of Centro Hospitalar Vila Nova de Gaia/Espinho, Vila Nova de Gaia, Portugal on 19 April 2018 (No. 57/2018–1); Centro Hospitalar do Baixo Vouga, Aveiro, Portugal on 23 May 2018 (No. 17–01–2018); and Centro Hospitalar Entre Douro e Vouga, Santa Maria da Feira, Portugal on 25 September 2018 (CA-0703/18). Written informed consent was obtained from all participants. In addition, the study followed the STROBE guidelines for observational studies.^24^ The protocol is available and registered at clinicaltrials.gov (NCT03499730). All methods were performed following the relevant policies and regulations.

#### Study design

This is a secondary analysis of a multicentre prospective observational cohort study.^25^

### Setting

Three ambulatory surgery units serving different populations from the north and centre of Portugal participated in the study, enrolling patients from 12 September 2018, to 5 March 2020.

### Study outcomes

The primary outcome of the study was postoperative pain, assessed at 24 h, 7 days, 3 months and 1 year after surgery.

The secondary outcomes were patient satisfaction and recovery.

### Participants

All adult patients scheduled for ambulatory open inguinal hernia repair were screened for eligibility on the day of surgery. Exclusion criteria included serious psychiatric disorders (alcoholism, patients under psychiatric medication other than antidepressants), illiteracy or poor understanding of the Portuguese language, history of chronic pain under opioid treatment, recurrent hernia, bilateral surgery and refusal. If eligible, the study protocol was explained and written consent obtained.

### Study protocol

All participants underwent a standard pre-operative anaesthetic assessment several weeks before surgery, independent of the study. On the morning of the surgery, patients were asked to complete a self-report pre-operative questionnaire including demographic data, and pre-operative pain and anxiety scales. Surgery was performed under standardised general anaesthesia [fentanyl 2 to 2.5 μg kg^−1^ intravenously (i.v.), propofol 1 to 2 mg kg^−1^ i.v. and sevoflurane through a laryngeal mask] or spinal anaesthesia (8 to 9 mg of 0.5% hyperbaric bupivacaine intrathecal). The type of anaesthesia (general or spinal) and dose of pre-operative i.v. supplementary midazolam (from none to any dosage) were chosen by the attending anaesthesiologist. All patients received wound infiltration with 10 ml of ropivacaine 0.75%; intravenous paracetamol 1 g and ketorolac 30 mg i.v. Rescue analgesia in the recovery room was provided as necessary with tramadol 2 mg kg^−1^ i.v.

### Data collection

Postoperative pain was the primary outcome variable, assessed through an 11-point NRS at 24 h, 7 days, 3 months and 1 year after surgery.

The primary independent variable was pre-operative anxiety, assessed using an 11-point ANRS (0 to 10) and the SFQ (0 to 80). Our ANRS consisted of numbers from zero to 10 aligned horizontally, introduced by the following written instructions: ‘On a scale from zero to 10, with zero meaning “no anxiety” and 10 meaning “the maximum anxiety you can imagine”, please mark the level of anxiety you feel at this moment’.

Other possible predictors of postoperative pain were pre-operative pain, age, sex, BMI, smoking status, employment status, education level, ambulatory status, American Society of Anesthesiologists physical status (ASA), chronic benzodiazepine usage and surgical and anaesthetic techniques. Pre-operative pain was assessed using an 11-point NRS (0 to 10) and the Pain Severity Scale from the Portuguese-validated version of the Brief Pain Inventory.^26^ Data such as age, weight, height, sex, smoking status, employment status, education level and pre-operative pain were self-reported in the pre-operative questionnaire. Peri-operative data were recorded in the theatre by the anaesthesiologist.

As a secondary aim of this study, pre-operative anxiety was evaluated for its association with other predictive conditions: pre-operative pain, age, sex, BMI, smoking status, employment status, education level, ambulatory unit, ASA and chronic benzodiazepine usage.

Follow-up telephone interviews were carried out by two blinded investigators at four different follow-up points: 24 h, 7 days, 3 months and 1 year after surgery. Data included postoperative pain, analgesic consumption, patient satisfaction NRS (0 to 10), adverse events and Global Surgery Recovery index (GSRI) (0 to 100%). The GSRI is a self-perceived measure of recovery that compares activity before and after surgery.^27^

We considered that patients’ sex, age, pre-operative inguinal pain, ambulatory unit, working status, literacy, type of anaesthesia and midazolam dose were potential confounders of the effect of anxiety on pain, as they could affect both the pre-operative anxiety level and postoperative outcomes.

### Study size

This is a secondary analysis of data from a previously conducted study.^25^ The sample size (*n* = 296) was initially calculated based on the primary outcome of the main research.

### Statistical analysis

Statistical analyses were performed using IBM SPSS (version 27), and the significance level was set at *P* = 0.05. Categorical variables are presented as *n* (%) and were compared using the Pearson Χ^2^ test. Pain and anxiety scores were used as continuous variables. All continuous variables were normally distributed as skewness varied between -1 and 1; these data are presented as mean ± SD and compared using a two-sided Student's *t*-test or one-way analysis of variance (ANOVA). Correlations between the anxiety scales were tested with the Pearson correlation coefficient. All tests are referenced in the tables. Missing data were not replaced, and outliers were not removed.

For logistic regression, age, BMI, anxiety, pain, satisfaction and GRSI scores were transformed into categorical variables. Pre-operative pain was defined as NRS ≥4, moderate postoperative pain as 4≤ NRS 7 < , and severe postoperative pain as NRS ≥ 7. For age, groups were defined using the terciles; for BMI, the threshold for obesity was determined according to the WHO criteria.

Receiver operating characteristics (ROC) curve analysis^28^ was performed using Youden's index^29^ to evaluate how well the anxiety scales predict postoperative pain and to identify the optimal cutoff points that distinguish between patients with and without clinically relevant postoperative pain (e.g. moderate or severe pain). These cutoff values were used to define anxiety subgroups. Difference between ROC AUC was assessed comparing the 95% confidence intervals (95% CIs).

To confirm the predictive validity of the anxiety scales on outcomes, simple and multiple logistic regression models were conducted, adjusting for sex, age, ambulatory unit, pre-operative pain, literacy, working status, type of anaesthesia (spinal vs. general) and midazolam dose administered pre-operatively. Multiple logistic regression models were run to identify independent predictors of high pre-operative anxiety; variables were included if *P* < 0.1 in univariate analysis, and backward stepwise selection was applied. For binary outcomes, estimates of adjusted odds ratios (adjOR) (95% CIs) are presented. Model calibration was assessed using the Hosmer-Lemeshow Test.

## Results

### Participants

**Fig. 1:**
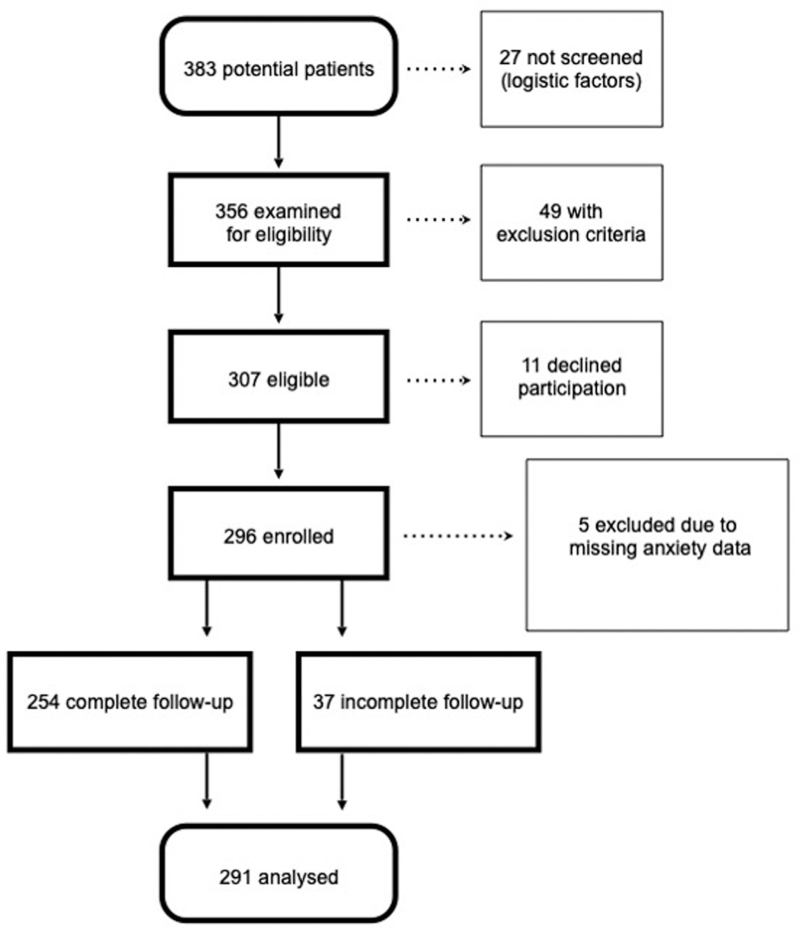
Participant flowchart.

Two hundred and ninety-six patients were enrolled between 12 September 2018 and 5 March 2020; 254 completed the 12-month follow-up, and 291 were analysed for anxiety data. The study flow chart is shown (Fig. 1). Two hundred and sixty men and 31 women were included; their characteristics are presented in Table 1.

**Table 1:** Patient characteristics

	Total *n* = 291	Missing *n* (%)
Age (years)	57 ± 13	0 (0)
BMI (kg m^−2^)	25.6 ± 2.8	4 (1)
Sex; male	260 (90%)	0 (0)
Smoker	54 (19%)	0 (0)
Chronic benzodiazepine usage	20 (7%)	1 (0)
Working status		2 (1)
Active	169 (59%)	
Unemployed	24 (8%)	
Retired	96 (33%)	
Education		0 (0)
<5 years	160 (55%)	
5 to 9 years	58 (20%)	
10 to 12 years	54 (19%)	
University	19 (6%)	
ASA		0 (0)
1	58 (20%)	
2	224 (77%)	
3	9 (3%)	
Pre-operative pain		
NRS	1.9 ± 2.4	1 (0)
≥4	68 (23%)	
Type of anaesthesia		1 (0)
Spinal	139 (48%)	1 (0)
General	151 (52%)	
Midazolam dosage (mg)	1.7 ± 1.1	0 (0)

Data are presented as *n* (%) or mean ± SD.

NRS, Numeric Rating Scale.

Twelve patients (4%) scored zero on both anxiety scales. An additional 42 patients (15%) scored zero only on the ANRS, while 19 (7%) scored zero only on the SFQ. There was a significant positive correlation between the two scales (*r* = 0.531, *P* < 0.001), with the ANRS showing the strongest correlation with the short version of the SFQ (*r* = 0.571, *P* < 0.001).

Among the SFQ sub-dimensions, scores for fear of short-term consequences (items 1 to 4) were significantly higher than those for fear of long-term consequences (items 5 to 8) (*P* < 0.001). Detailed results for total scores, sub-dimensions and individual items of the SFQ are presented in Table 2.

**Table 2:** Results of different anxiety measures

	Mean ± SD	Range
SFQ1: Fear of Operation	3.0 ± 2.5	0 to 10
SFQ2: Fear of Anaesthesia	2.7 ± 2.6	0 to 10
SFQ3: Fear of Pain	3.7 ± 2.6	0 to 10
SFQ4: Fear of Side-effects	2.9 ± 2.5	0 to 10
SFQ5: Fear of Health deterioration	2.3 ± 2.6	0 to 10
SFQ6: Fear of Failed operation	2.0 ± 2.4	0 to 10
SFQ7: Fear of Incomplete recovery	2.3 ± 2.5	0 to 10
SFQ8: Fear of Long rehabilitation	2.8 ± 2.7	0 to 10
SFQ-short	12.3 ± 8.7	0 to 38
SFQ-long	9.3 ± 8.9	0 to 40
SFQ total	21.6 ± 15.8	0 to 77
ANRS	3.7 ± 2.5	0 to 10

Data are presented as mean ± SD and range.

### Performance of anxiety scales

We evaluated the performance of the ANRS and the SFQ (including its short and long subscales) in predicting postoperative pain, defined as an NRS score of at least 4, at four different follow-up time points: 24 h, 7 days, 3 months and 1 year after surgery. Overall, the predictive ability of all scales was poor, with the SFQ showing better results than the ANRS at most time points, although these differences were not statistically significant for the ROC AUC. When comparing the sub-dimensions of the SFQ, the short version demonstrated slightly better predictive values than the long version across all time points. ROC AUC with 95% CI are presented in Table 3.

**Table 3:** Performance of anxiety scales to predict postoperative pain

	ANRS ≥ 5		SFQ ≥ 21 / 25	
		
	AUC (95% CI)	Sensitivity/specificity	AUC (95% CI)	Sensitivity/specificity
24 h	0.571 (0.496 to 0.647)	0.429/0.685	0.630 (0.558 to 0.702)	0.533/0.652
7 days	0.610 (0.542 to 0.678)	0.459/0.697	0.639 (0.573 to 0.706)	0.589/0.672
3 months	0.607 (0.519 to 0,696)	0.542/0.641	0.616 (0.5 to 0.698)	0.542/0.641
1 year	0.651 (0.565 to 0.737)	0.583/0.652	0.645 (0.554 to 0.737)	0.625/0.657

ANRS, Anxiety Numeric Rating Scale, cutoff ≥ 5; AUC, area under the curve; SFQ, Surgical Fear Questionnaire, cutoff ≥ 21 for acute pain and 25 for chronic pain.

The analysis of Youden's index indicated that an ANRS cutoff score of four was optimal for predicting all postoperative pain outcomes. For the SFQ, a cutoff score of 20 best predicted acute pain, while a score of 24 was more accurate for predicting chronic pain. Sensitivity and specificity for these cutoffs are presented in Table 3. Due to variability in optimal cutoff points across outcomes, the SFQ short-term and long-term subscales were not further analysed. On the basis of these cutoffs, the cohort was divided into two groups (high and low anxiety) according to their pre-operative SFQ and ANRS scores.

Table 4 summarises the pain scores for the 274 patients who completed at least one postoperative follow-up. The type of anaesthesia, smoking status and postoperative analgesic consumption were comparable across the high and low-anxiety subgroups.

**Table 4:** Follow-up data by anxiety subgroups

	SFQ 0-20 *n* = 143	SFQ 21-80 *n* = 131	*P*	ANRS 0–4 *n* = 167	ANRS 5-10 *n* = 107	*P*	Missing *n* (%)
At 24 h							0 (0)
Pain NRS	3.3 ± 1.9	4.5 ± 2.0	<0.001^a^	3.6 ± 2.0	4.3 ± 2.0	0.003^a^	
Pain ≥4	85 (59%)	99 (76%)	0.005^b^	105 (63%)	79 (74%)	0.060^b^	
Pain ≥7	22 (15%)	39 (30%)	0.004^b^	30 (18%)	31 (29%)	0.033^b^	
At 7 days							3 (1)
Pain NRS	2.9 ± 1.6	3.8 ± 1.7	<0.001^a^	3.2 ± 1.6	3.6 ± 1.8	0.023^a^	
Pain ≥4	60 (42%)	87 (67%)	<0.001^b^	80 (48%)	68 (65%)	0.008^b^	
Pain ≥7	3 (2%)	10 (8%)	0.030^b^	5 (3%)	8 (8%)	0.084^b^	
Satisfaction = 10	87 (61%)	55 (43%)	0.003^b^	84 (51%)	59 (56%)	0.397^b^	4 (1)
At 3 months							4 (1)
Pain NRS	0.9 ± 1.4	1.5 ± 2.1	0.002^a^	1.0 ± 1.4	1.5 ± 2.2	0.015^a^	
Pain ≥4	17 (12%)	31 (24%)	0.011^b^	22 (13%)	26 (25%)	0.018^b^	
Pain ≥7	2 (1%)	9 (7%)	0.021^b^	3 (2%)	8 (8%)	0.020^b^	
Satisfaction = 10	99 (70%)	71 (55%)	0.008^b^	107 (65%)	64 (60%)	0.457^b^	3 (1)
GRSI	92 ± 13	82 ± 20	<0.001^a^	89 ± 14	85 ± 20	0.025^a^	3 (1)
At 1 year							16 (6)
Pain NRS	0.7 ± 1.6	1.4 ± 2.2	0.005^a^	0.7 ± 1.7	1.5 ± 2.3	0.003^a^	
Pain ≥4	16 (12%)	33 (27%)	0.003^b^	20 (13%)	28 (28%)	0.003^b^	
Pain ≥7	4 (3%)	16 (13%)	0.003^b^	6 (4%)	13 (13%)	0.007^b^	

Values are *n* (%) or mean ± SD. SFQ, Surgical Fear Questionnaire; ANRS, Anxiety Numeric Rating Scale; GSRI, Global Surgery Recovery Index.

^a^*t*-test.

^b^Pearson Χ^2^ test.

Logistic regression analysis confirmed that higher pre-operative anxiety was associated with worse postoperative outcomes. Table 5 presents the crude and adjORs for postoperative pain, patient satisfaction and recovery based on the ANRS and SFQ scores.

**Table 5:** Predictive value of high anxiety on postoperative outcomes

Outcome	Predictor	OR (95% CI)	*P* ^*^	adjOR (95% CI)^a^	*P* ^*^
24 h PAIN ≥4	SFQ	**2.111 (1.255 to 3.550)**	**0.004**	**1.751 (1.002 to 3.060)**	**0.048**
	ANRS	1.666 (0.977 to 2.840)	0.057	1.385 (0.772 to 2.484)	0.272
7 d PAIN ≥4	SFQ	**2.831 (1.723 to 4.651)**	**<0.001**	**2.703 (1.578 to 4.630)**	**<0.001**
	ANRS	**1.976 (1.195 to 3.267)**	**0.007**	1.602 (0.925 to 2.773)	0.092
3m PAIN ≥4	SFQ	**2.289 (1.197 to 4.377)**	**0.011**	**2.143 (1.072 to 4.283)**	**0.029**
	ANRS	2.124 (1.130 to 3.992)	0.019	1.582 (0.786 to 3.186)	0.201
Satisfaction = 10	SFQ	**0.511 (0.310 to 0.841)**	**0.008**	**0.546 (0.318 to 0.939)**	**0.028**
	ANRS	0.826 (0.499 to 1.367)	0.457	0.943 (0.537 to 1.656)	0.839
GSRI ≥90	SFQ	**0.300 (0.177 to 0.507)**	**<0.001**	**0.294 (0.168 to 0.517)**	**<0.001**
	ANRS	0.750 (0.451 to 1.249)	0.270	0.938 (0.536 to 1.643)	0.824
1 y PAIN ≥4	SFQ	**2.674 (1.387 to 5.158)**	**0.003**	**2.414 (1.184 to 4.921)**	**0.014**
	ANRS	**2.627 (1.385 to 4.984)**	**0.003**	**2.071 (1.026 to 4.180)**	**0.042**

Predictive value of high anxiety on postoperative outcomes. Odds ratio (95% confidence intervals); ^a^adjusted for sex, age, ambulatory unit, pre-operative pain, literacy, working status, type of anaesthesia and midazolam dose. ANRS, Anxiety Numeric Rating Scale, using five as cutoff; SFQ, Surgical Fear Questionnaire, using 21 as cutoff. Statistically significant results are in bold. ^*^Likelihood ratio test.

### Risk factors for high pre-operative anxiety

Table 6 presents the anxiety scores for the different patient subgroups. The SFQ detected differences in anxiety based on age, sex, BMI, employment status and pre-operative pain, but the ANRS was only sensitive in detecting differences based on pre-operative pain.

**Table 6:** Anxiety in different patient subgroups

		SFQ	*P*	ANRS	*P*
Age	18 to 48	26.0 ± 18.0	**0.005** ^**a**^	3.6 ± 2.1	0.704^a^
	49 to 66	21.5 ± 15.8		3.8 ± 2.7	
	≥ 67	17.2 ± 11.8		3.5 ± 2.3	
Sex	Male	20.9 ± 15.7	**0.027** ^**b**^	3.6 ± 2.5	0.094^b^
	Female	27.7 ± 15.4		4.4 ± 2.7	
BMI	< 30	22.4 ± 16.1	**0.023** ^**b**^	3.7 ± 2.5	0.265^b^
	≥ 30	14.2 ± 11.6		3.1 ± 2.3	
Education	< 9 years	20.0 ± 15.3	0.055^b^	3.7 ± 2.7	0.894^b^
	≥9 years	23.5 ± 16.2		3.7 ± 2.2	
Employment	Working	24.2 ± 16.7	**0.002** ^**a**^	3.9 ± 2.5	0.219^a^
	Unemployed	19.6 ± 12.9		3.6 ± 2.8	
	Retired	17.1 ± 13.3		3.3 ± 2.8	
Smoker	Yes	23.9 ± 19.2	0.246^b^	3.9 ± 2.8	0.391^b^
	No	21.1 ± 14.9		3.6 ± 2.4	
Chronic benzodiazepine	Yes	21.9 ± 11.6	0.927^b^	3.5 ± 2.5	0.680^b^
	No	21.6 ± 16.1		3.7 ± 2.5	
Pre-operative pain	< 4	19.9 ± 15.3	**<0.001** ^**b**^	3.3 ± 2.4	**<0.001** ^**b**^
	≥ 4	27.9 ± 16.6		5.0 ± 2.5	

Data are given as mean ± SD.

ANRS, Anxiety Numeric Rating Scale; SFQ, Surgical Fear Questionnaire.

Statistically significant results are in bold.

^a^One-way ANOVA.

^b^*t*-test.

Multivariable logistic regression identified younger age, female sex, BMI less than 30 and the presence of pre-operative pain as independent predictors of high pre-operative anxiety (Table 7). In contrast, being an active worker was not a significant predictor after adjusting for other variables. In addition, smoking status, ASA classification, chronic benzodiazepine use and literacy level were not significantly associated with pre-operative anxiety.

**Table 7:** Independent predictors of high anxiety (SFQ ≥ 21)

Predictor	OR (95% CI)	*P* ^*^	adjOR (95% CI)^a^	*P* ^*^
Age (years)	**0.975 (0.957 to 0.994)**	**0.009**	**0.969 (0.949 to 0.989)**	**0.002**
Female sex	**2.697 (1.183 to 6.148)**	**0.014**	2.262 (0.949 to 5.394)	0.060
BMI <30	**3.125 (1.111 to 8.788)**	**0.020**	**3.109 (1.077 to 8.973)**	**0.025**
Pre-operative pain ≥4	**2.364 (1.340 to 4.173)**	**0.003**	**1.973 (1.065 to 3.656)**	**0.029**

Odds ratio (95% confidence intervals).

SFQ, Surgical Fear Questionnaire.

Statistically significant results are in bold. ^a^Adjusted for sex, age, BMI and pre-operative pain. ^*^Likelihood ratio test.

## Discussion

To our knowledge, this is the first study to directly compare the SFQ and the ANRS in assessing pre-operative anxiety. The SFQ demonstrated greater sensitivity than the ANRS, as it more effectively identified differences in anxiety based on age, sex, BMI, employment status and preoperative pain. In contrast, the ANRS was only associated with pre-operative pain.

Both anxiety scales showed some ability to predict postoperative pain, with the SFQ performing slightly better than the ANRS across most follow-up points. The optimal cutoff score for predicting pain was four for the ANRS and 20 and 24 for the SFQ when predicting acute and chronic pain, respectively.

Higher pre-operative SFQ scores were significantly associated with worse postoperative outcomes, including increased postoperative pain, lower patient satisfaction and worse recovery. These associations remained significant after adjustment for demographic and clinical factors.

Finally, in our population, the independent predictors of high pre-operative anxiety were younger age, female sex, a BMI below 30 and the presence of pre-operative pain. The anxiety levels observed in our study population were comparable to those reported in other ambulatory surgery cohorts.^18,30^

Patients reported the greatest fear of postoperative pain, followed by concerns about the surgery itself, potential side effects, prolonged rehabilitation and, to a lesser extent, anaesthesia. This contrasts with findings from one of the earliest surveys on pre-operative anxiety,^31^ in which anaesthesia was the most feared aspect and postoperative pain was considered least important. However, more recent studies^32–35^ have similarly shown that patients tend to fear surgery more than anaesthesia, likely a reflection of the growing trust in anaesthetic care, owing to significant advancements in the speciality.

The SFQ appears to be a more effective tool than the ANRS for identifying patients at risk of worse postoperative outcomes related to high pre-operative anxiety. In our study, the SFQ demonstrated better discriminative performance across pain, satisfaction and recovery outcomes. It also detected anxiety differences across key demographic and clinical variables, including age, sex, BMI, employment status and pre-operative pain. Despite this, the ANRS still showed acceptable performance and offers important advantages in practical settings: it is more straightforward, quicker to administer and well suited in busy ambulatory environments. The ANRS can be easily implemented as part of a routine verbal pre-operative interview without requiring additional paperwork or literacy.^20^

Although the SFQ was initially validated in Portuguese-speaking female in-patients undergoing major surgery, our study population differed considerably, primarily male patients scheduled for ambulatory procedures. In addition, the original validation involved patients who completed the SFQ at home in the week leading up to surgery. In contrast, in our study, the questionnaire was administered on the day of surgery. Optimal time for anxiety screening has not been defined and despite these differences in patient demographics and timing of assessment, the SFQ demonstrated comparable performance, suggesting it is a stable and reliable tool across varied surgical populations and settings.

ROC analysis showed that both anxiety scales had poor discriminative power for predicting postoperative pain, with low sensitivity and specificity values. Nonetheless, the SFQ consistently outperformed the ANRS. The optimal cutoff scores were close to the mean values observed: four for the ANRS and 20 for the SFQ. These findings align with previous research, which has proposed similar cutoffs for the ANRS,^21,22^ while highlighting a gap in the SFQ literature,^18^ where no clinical thresholds have been established. Our proposed cutoffs offer guidance for both clinical use and future research, which could explore the use of combined assessment tools integrating anxiety with other variables in order to better predict postoperative pain.

This study has several notable strengths. First, its multicentre design across three Portuguese ambulatory surgery units enhances the generalisability of the findings to similar clinical settings. The large and well defined cohort of nearly 300 patients undergoing a uniform procedure – open inguinal hernia repair – ensures internal consistency and methodological robustness. The high follow-up rate at 12 months further strengthens the validity of the longitudinal outcomes. A significant advantage of this study is the direct, head-to-head comparison of two anxiety assessment tools – the SFQ and the ANRS – within the same patient population, allowing for a meaningful evaluation of their predictive performance. Using objective statistical methods, including ROC analysis, Youden's index and multivariable logistic regression, adds rigour to the analysis.

### Limitations

The sample was predominantly male, reflecting the epidemiology of inguinal hernias. Still, this sex imbalance may limit the applicability of findings – particularly the anxiety cutoff values – to female patients, who reported higher SFQ scores. In addition, the focus on a single surgical procedure enhances homogeneity but may reduce the generalisability of results to other types of surgery or more complex patient populations. Anxiety was assessed on the day of surgery, which may not capture fluctuating levels of distress in the days leading up to the procedure, as would be possible with earlier assessments. Moreover, although the SFQ was previously validated in Portuguese, it was done so in a different patient group – female inpatients undergoing major surgery – raising questions about its applicability in the predominantly male, ambulatory context of this study. Other limitations include the exclusion of patients with psychiatric disorders or chronic opioid use, and the lack of assessment of psychosocial variables such as depression or coping style, which may also influence both anxiety and pain perception.

Future research on this topic could aim to validate the SFQ and the ANRS in other operative contexts, to determine the ideal cutoff for different groups of patients, or to define the optimal time for pre-operative anxiety screening.

## Conclusion

Pre-operative anxiety assessment is valuable for anaesthesiologists to identify patients at an increased risk of postoperative complications, including pain, dissatisfaction and delayed recovery. The ANRS, with a suggested cutoff of 4, demonstrated acceptable predictive value for postoperative pain and offered the advantage of being quick, simple and accessible, even for patients with low literacy. The SFQ, while slightly more complex, provided superior discrimination of high-anxiety patients and was more sensitive to demographic and clinical differences. Although both instruments showed suboptimal discriminative power for postoperative pain, we recommend incorporating objective anxiety assessment into routine clinical pre-anaesthetic evaluation, while waiting for the development of better assessment tools that might combine anxiety with other variables.
